# Editorial: Sulfation Pathways—There and Back Again

**DOI:** 10.3389/fmolb.2022.912700

**Published:** 2022-05-16

**Authors:** Jon Wolf Mueller, Abby C. Collier, Tarsis F. Gesteira

**Affiliations:** ^1^ Institute of Metabolism and Systems Research, University of Birmingham, Birmingham, United Kingdom; ^2^ Faculty of Pharmaceutical Sciences, University of British Columbia, Vancouver, BC, Canada; ^3^ College of Optometry, University of Houston, Houston, TX, United States

**Keywords:** PAPS synthase, sulfation pathways, protein folding/stability/aggregation, conjugate analytics/mass spectrometry, sulfo-metabolite synthesis/analytics

**FIGURE 1:**
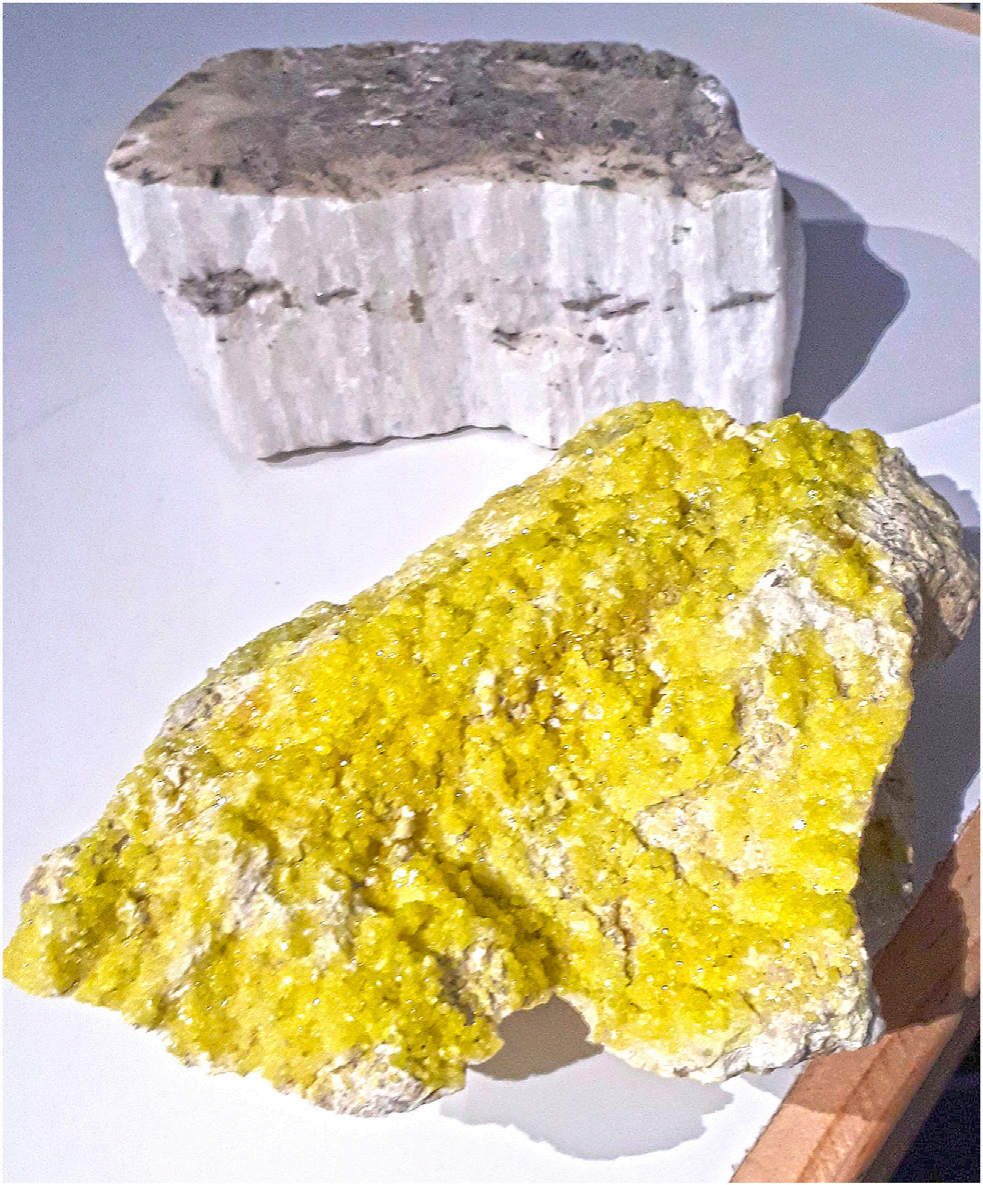
Sulfur and a rock of sulfate. A lump of elementary sulfur featuring many shades of sulfuric yellows (front) and a rock of gypsum (rear), also known as CaSO_4_ • 2 H_2_O. Both specimens were kindly provided by the Lapworth Museum of Geology, University of Birmingham, for an outreach event (to JWM).

Sulfation Pathways are understood as the oxidative branch of sulfur metabolism (Günal et al., 2019). Core to sulfation pathways is sulfate activation, the transfer of sulfate to biological acceptor molecules and its dynamic cleavage in a spatially and temporally specific manner. The biochemical problem of sulfate activation is evident even in geological specimens–gypsum may be observed in orthopedic casts, an example of the highly inert sulfate ion (Figure 1). A significant amount of energy is needed to turn biological sulfate into PAPS (3′-phospho-adenosine-5′-phospho-sulfate), the active sulfate form.

The many and diverse sulfation acceptor molecules in biology dictate the molecular functions of ubiquitous sulfation pathways. Recent publications have highlighted doubly sulfated steroids (Lightning et al., 2021) as well as sulfo-conjugated vitamin D species (Jenkinson et al., 2022). This Research Topic focused on *Advances in Sulfation Pathways Research* features nine research articles and three reviews, authored by 63 individual scientists, significantly advancing the field of Sulfation Pathways, highlighted within the following paragraphs:

Research into sulfation pathways always relied on state-of-the-art analytical methods. Hence, we are pleased to present novel mass-spectrometry-based ways of profiling sulfated metabolites in urine by Fitzgerald et al. Developing mass spectrometry methods is impossible however without having the respective pure compounds readily at hand. In this regard, Alshehri et al. report a novel sulfation strategy to prepare steroid sulfates and isotopically labeled variations thereof, certainly an innovation with great importance for the field. Whenever coming from a genomic viewpoint, it may be necessary to characterize a sulfation enzyme with regards to its activity. With this in mind, Sun et al. have prepared a manuscript on optimized sulfotransferase assays.

Sulfation impacts greatly on steroidogenesis–sulfated steroids may represent transport forms and/or modulate downstream processing (Foster and Mueller, 2018). Two studies by Pavlič et al. and De Almeida Da Costa et al. independently provide experimental evidence that endometrial tissues can generate estrogens from circulating steroid sulfates. Sulfation of steroids predominantly occurs within the cytosol and sulfated steroids then need dedicated transporters to enter circulation and for the uptake into target tissues. Karakus et al. provide an in-depth study about the steroid sulfate uptake transporter SOAT (SLC10A6) in adipose tissue that illustrates this well.

When studying sulfated steroids, not only are circulating steroids important, but also sulfated neuro-steroids. This Research Topic features two review articles about steroid sulfation in neurodegenerative diseases by Vitku et al. and during neurodevelopment by Clarke et al. Fascinating topics that will enable exciting discoveries in future, with great potential for the development of novel therapeutics.

Aspects of sulfation pathways are highly conserved in multicellular organisms. Hence, the review article by Igreja and Sommer about sulfation pathways in nematode development will be of great interest to the community–much is known about the genetics of these pathways, however a lot remains to be uncovered from the metabolism side.

One part of sulfation pathways that differs greatly between species is degradation of the remnants of PAPS-dependent sulfation. 3′-Phospho-adenosine-5′-phosphate seems to be a waste product in humans–it however represents important signaling properties in plants. Ashykhmina et al. studied the divergent functions of this nucleotide in stress signaling, when encountered in different cellular compartments. It remains to be seen, if any of these signaling propensities would also be discovered in human sulfation pathways.

From a synthetic biology perspective, some sulfation proteins have been characterized in their folding and protein stability, reviewed here (Brylski et al., 2019), following on from which Brylski et al. constructed a folding sensor that reports protein integrity *via* Förster resonance energy transfer. This elegant advance allowed measurement of protein unfolding within intact cells. With this approach, Brylski et al. established that cellular ATP levels determine the stability of a nucleotide kinase. The same team of researchers also studied the stability and aggregation behavior of disease-related protein variants of the highly conserved enzyme PAPSS2 (Brylski et al.).

We look towards a bright future in Sulfation Pathways research. New developments may bring insights in novel or overlooked sulfated metabolites. Linking sulfation pathways with other metabolic pathways may also be highly interesting–such as the study of two Golgi-residing heparan sulfate sulfotransferases that cooperatively act on their substrate (Gesteira et al., 2021). By investigation of patients with rare genetic mutations or by using omics and big data approaches, we also may be able to link sulfation pathways to other biological themes; and linking steroid sulfation to adrenal tumors (Mueller et al., 2021) is a first step towards this goal.

There were dedicated meetings on sulfation pathways back in the 1990s. Due to renewed interest and significant advances in the field, such meetings started again. SUPA meetings took place in Greifswald, Germany, in 2015; then continued in Birmingham, United Kingdom, in 2017, and Castle Rauischholzhausen, Germany, in 2019. Accompanying Research Topics on sulfation pathways were published in 2016 (Mueller and Muller, 2016) and 2018 (Mueller and Foster, 2018). We are delighted to present the current Research Topic, looking towards the SUPA 2023 meeting that is being organized already.
